# Minimally-Invasive Neural Interface for Distributed Wireless Electrocorticogram Recording Systems

**DOI:** 10.3390/s18010263

**Published:** 2018-01-17

**Authors:** Sun-Il Chang, Sung-Yun Park, Euisik Yoon

**Affiliations:** 1 Apple Incorporated, Cupertino, CA 95014, USA; sunil_chang@apple.com; 2 Department of Electrical Engineering and Computer Science, University of Michigan, Ann Arbor, MI 48109, USA; sungyun@umich.edu

**Keywords:** Electrocorticogram (ECoG), low-power, low-noise, neural recording, push-pull double-gated amplifier, intra-skin communication (ISCOM), neural interface

## Abstract

This paper presents a minimally-invasive neural interface for distributed wireless electrocorticogram (ECoG) recording systems. The proposed interface equips all necessary components for ECoG recording, such as the high performance front-end integrated circuits, a fabricated flexible microelectrode array, and wireless communication inside a miniaturized custom-made platform. The multiple units of the interface systems can be deployed to cover a broad range of the target brain region and transmit signals via a built-in intra-skin communication (ISCOM) module. The core integrated circuit (IC) consists of 16-channel, low-power push-pull double-gated preamplifiers, in-channel successive approximation register analog-to-digital converters (SAR ADC) with a single-clocked bootstrapping switch and a time-delayed control unit, an ISCOM module for wireless data transfer through the skin instead of a power-hungry RF wireless transmitter, and a monolithic voltage/current reference generator to support the aforementioned analog and mixed-signal circuit blocks. The IC was fabricated using 250 nm CMOS processes in an area of 3.2 × 0.9 mm^2^ and achieved the low-power operation of 2.5 µW per channel. Input-referred noise was measured as 5.62 µV_rms_ for 10 Hz to 10 kHz and ENOB of 7.21 at 31.25 kS/s. The implemented system successfully recorded multi-channel neural activities in vivo from a primate and demonstrated modular expandability using the ISCOM with power consumption of 160 µW.

## 1. Introduction

The neural interfaces enable us to build a direct communication pathway between the human brain and external world, through which we can monitor neural activities, supplement the nervous system using a neuroprosthetic device, and even assist and augment human cognitive or sensory functions by brain-computer interface (BCI). In practice, the brain’s spontaneous or stimulated activities should be closely monitored to provide the important neurofeedback for rehabilitation or for parts of evaluation and titration of therapy in neurological disorders such as epilepsy, Parkinson’s disease, strokes, etc. The same neural interface systems can be also useful tools for neuroscience studies in exploring nervous systems and understanding brain circuits and their complex activities and connectivity [1,2].

The development of neural interface systems gives a trade-off between signal fidelity and subject safety, depending on where and how the electrodes are being deployed and located, e.g., electroencephalogram (EEG), electrocorticogram (ECoG), or single unit action potential (SUAP). The SUAP gives us the most accurate neural activity information; however, the system must be severely invasive due to the necessity of using micro-machined electrodes implanted to reach single neurons [3], and may lead to tissue reaction. The EEG potential can be obtained on the surface of the scalp without penetration of a cranium, allowing a non-invasive system. However, the bio-information achieved by the EEG system is limited in temporal and spatial resolution because it can only collect the ensemble activities of neuron groups [4]. On the other hand, the ECoG recently gains its attention as a compromised solution for balancing between signal fidelity and safety [5,6,7,8,9,10,11,12,13]. The effectiveness of the ECoG recording to decode neural signals by using spectrograms has been demonstrated in recent articles [12,14,15]. The ECoG system can record brain activities on the surface of the cortex, not penetrating brain. Therefore, the ECoG system is less invasive than the SUAP recording system, and the recorded signals can provide more abundant bio-information than surface EEG signals. In addition, the recent observation of spikes and field potentials of the cortical neurons from the brain surface has been reported, which makes the surface recording more effective method [16].

The ECoG brain signals typically have a low amplitude (~100 μV) and a relatively low bandwidth (<500 Hz). Thus, it is important to provide low-noise signal amplification and digitization for the given signal range and bandwidth. In addition, in order to increase the temporal and spatial resolution for useful clinical and research applications, multiple locations need to be simultaneously monitored up to >200 electrodes over the large area of the brain [17]. Although much better than the SUAP monitoring in terms of safety, ECoG is still regarded as an invasive method since it needs a craniotomy to place electrode arrays and implantable interface modules on top of cortex. It is desirable for the implanted system to be realized in a small form factor with an elongated lifetime so that it can operate for weeks or even for years once implanted. In order to prevent possible infection, it is essential to completely remove the transcutaneous connection between the implanted device and an external system by implementing a low-power wireless data transmission scheme in a minimized volume. Several groups have reported the implantable interface circuits and system level integration for ECoG/EEG recording with the on-chip spectral analysis function to provide the processed information with wireless telemetry for applications such as motion, epileptic seizure, and depression detection [5,6,7,8,9,18,19,20]. However, these approaches consume huge power and they can only allow a limited number of parallel recording channels at the given power budget and exhibit a bulky system volume. Recently, a 64-channel ECoG recording chip with an RF-based data transmission and power harvesting has been presented to overcome those limitations [5]. Although it exhibited the state-of-the-art power consumption (2.3 µW/Ch and 225 µW for total power consumption), there were drawbacks. It showed a significant channel-to-channel gain variation from their open-loop architecture which requires off-chip calibration upon start-up. Also, its front-end gain is limited due to the low supply voltage which was chosen to reduce power consumption [5]. Still, there are numerous technical challenges to be addressed in order to accomplish a reliable, fully-functional ECoG neural interface system. Low-power analog front-end circuits should be combined with wireless data telemetry in a small form factor with modular expandability for high channel counts.

We present a minimally invasive neural interface for multi-channel wireless ECoG recording by using the intra-skin communication (ISCOM) [8]. The wireless data transmission through ISCOM can provide multi-channel recording in a distributed system for a behaving animal. Moreover, the interface system can be all-in-one since it equips all necessary electronic components and units inside a small implantable platform, including a microelectrode array, an energy harvesting coil, and a rechargeable battery, an analog frontend, and an ISCOM wireless transceiver. In this paper, we focus on describing the design and implementation of low-power integrated circuits (ICs) suitable for the proposed wireless ECoG recording system. The implemented IC is the core unit that enables the recording of 16-channel of ECoG signals with high power-noise efficiency while providing a wireless ISCOM in combination of the custom-microfabricated flexible ECoG electrode array. This paper is organized as follows. Section 2 describes the overall architecture of the proposed system. Section 3 presents the details of IC design and implementation including a noise-power efficient double-gated push-pull preamplifier, a low-voltage successive approximation register analog-to-digital converter (SAR ADC), an on-chip programmable voltage/current reference generator, and a wireless ISCOM module. In Section 4, the design and fabrication of the flexible microelectrode array is given. In Section 5, the results of 16-channel in vivo ECoG measurements from a primate is presented, followed by the bench-top characterization of the fabricated IC. Finally, Section 6 concludes this paper.

## 2. System Architecture

### 2.1. Concept of Distributed Wireless ECoG Recording

**Figure 1:**
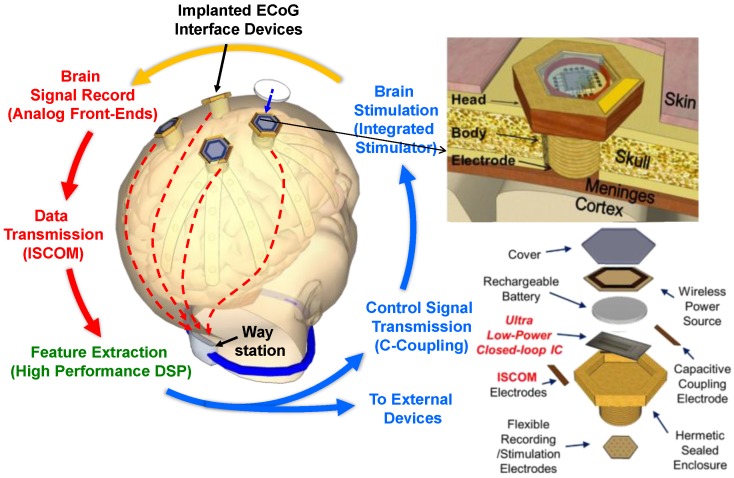
System concept for distributed wireless ECoG recordings.

Figure 1 shows the schematic diagram of the system implementation. Instead of high-risk craniotomy, a burr hole is opened and a flexible microelectrode array is inserted into the hole. A bolt-shaped implantable interface module is located inside the hole and covered by the skin. The proposed implementation can significantly reduce invasiveness by placing the electrode array over the dura matter, making an operation much simpler and less risky. The recorded ECoG signals are transmitted through the skin, instead of using the conventional wireless communication techniques, such as near or far field communication (RF or inductive coupling). Each bolt-shaped module sends the recorded signals through a low-power wireless ISCOM link to the way station that also resides on the body and relays the data to the external host. An external wireless link for the data exchange between the way station and the host (personal computer or workstation) can employ a standard wireless technology, e.g., WLAN, with a high data rate. The power consumption in the way station is not restricted because it does not need to be implanted inside the body; thus, a relatively large power source can be equipped in the way station. In addition, a digital signal processing (DSP) unit is implemented in the way station where we can cost-effectively perform feature extraction and classification with a modest power budget. One of the advantages in this scheme is that the algorithm for decoding neural signals in the way station can be easily upgraded and customized for a personalized solution. Each interface in the system has multiple components inside, consisting of a low-power, low noise IC for neural signal processing (recording, filtering, and digitization), a rechargeable battery, a wireless power source for the rechargeable battery, and the flexible ECoG and ISCOM electrodes at the bottom. All the components are hermetically sealed inside the enclosure.

### 2.2. Architecture of Neural Interface

**Figure 2:**
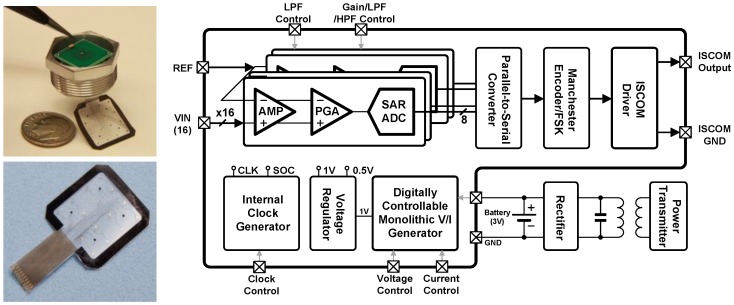
Photograph of the fabricated neural interface module and the flexible microelectrode array (**left**), the block diagram of integrated circuits in the module (**right**).

Figure 2 shows a photograph of the fabricated wireless neural interface module and flexible microelectrode array with an U.S. dime, and a block diagram of the integrated circuits inside the module. The volume of the neural module is ~1.3 cm^3^, and the microelectrode array has 16 recording electrodes and 4 reference electrodes with a large common ground within 18 × 18 mm^2^ in area. The integrated circuit also has 16 neural recording channels. Each channel contains a power-noise efficient preamplifier, a programmable gain amplifier, a bandpass filter (with a buffer), and a low power 8-bit SAR ADC as shown in Figure 2 (right). The digitized outputs from each channel are serialized, encoded, and transmitted wirelessly through the skin using an ISCOM driver which generates a charge-balanced (biphasic) output current. To reliably record ECoG signals regardless of their amplitudes, the gain in each channel is independently programmable (40–60 dB). The bandwidth and sampling rate (1~31.25 kS/s) are also adjustable for various sensing modalities to meet the wide range of applications. For system-level power optimization, the interface module utilizes two power supplies: 0.5 and 1.0 V for analog and digital blocks, respectively. These supply voltages are generated by the combination of low dropout regulators (LDOs, not shown here) and an embedded monolithic voltage/current reference generator operating at 1.5–3.0 V, provided by a rechargeable battery (used a coin-shaped Li-Ion rechargeable battery with a nominal output of 3 V). The control signals for SAR ADCs are internally generated and shared among 16 ADCs in the IC. All the system blocks operate with an on-chip clock generator. Also, digital programmability is provided to change the configuration for various users’ demands as well as to compensate for possible process variations. The following sections will explain the requirements, function, and design of each circuit block.

## 3. Integrated Circuits

In this section, the details of circuit design for essential building blocks in the proposed neural interface, such as preamplifiers, ADCs, reference generators, and ISCOM are described.

### 3.1. Noise-Power Efficient Double-Gated Push-Pull Preamplifier Using Quasi-Floating Body

In most of low-power, low-noise operational transconductance (*g_m_*) amplifiers (OTAs) in the previous works, the transconductance (*g_m_*) of the input transistors was maximized for high noise-power efficiency by operating them in the subthreshold region, the transconductance in the rest of the transistors was minimized by making them operate in the strong inversion region [21,22]. Recently, a push-pull topology (often called as a current reuse topology) was adopted to double the effective *g_m_* of the input transistor [23,24,25,26].

**Figure 3:**
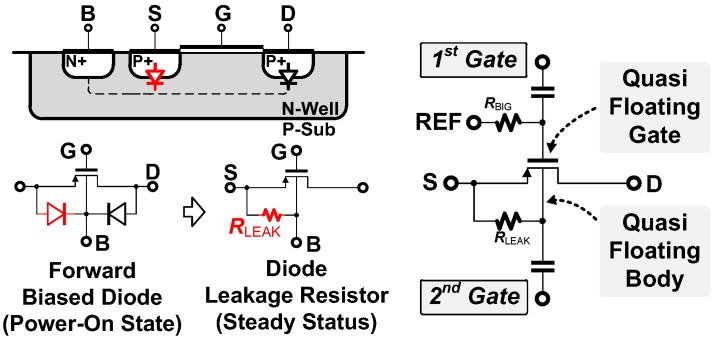
Double-gated PMOS transistor where the gate and body terminal are used as two inputs.

In this work, we have introduced a double-gated, push-pull preamplifier where the body terminal of the input transistors is used as the second gate to further improve the noise-power efficiency. Figure 3 illustrates the double-gated PMOS transistor implemented by using a quasi-floating body. In power-on state, the source potential initially connects to the supply voltage (*V_DD_* = 1 V), and the body is biased by a source-body junction diode which is weakly in forward-bias. As the potential of the body approaches to that of the source, the body is biased by a leakage current through a large parasitic resistive path (*R*_LEAK_ ≈ 50 GΩ) from the source, making the body can work as another input gate in the transistor. In the steady-state condition, the body leakage current is about 2 pA and the potential becomes ~900 mV, while maintaining a potential difference of 100 mV from the source terminal (according to our SPICE simulation). In the actual implementation, the quasi-floating bodies in the NMOS transistors are formed by using the triple wells from the given process technology. Both PMOS and NMOS transistors are isolated from rest of the transistors by using the double guard-rings in the layout.

**Figure 4:**
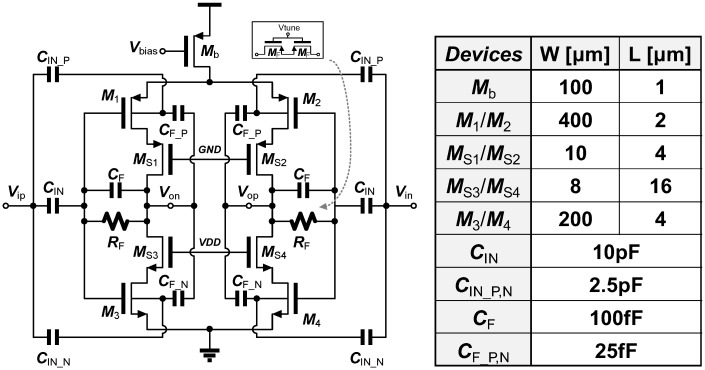
Schematic of the double-gated, push-pull preamplifier and the table of dimensions and values for the transistors and capacitors used in the preamplifier.

The overall schematic of the proposed preamplifier is shown in Figure 4. The effective transconductance of the OTA, *G_m_*, can be given by:

(1) $$G_{m}=\underset{Push-Pull\;Stage}{\underset{︸}{g_{m1}+g_{m3}}}+\underset{Quasi-Floating\;Body}{\underset{︸}{g_{mb1}+g_{mb3}}}=2g_{m}\left(1+\eta \right)\approx 2.4g_{m}$$

where *η* is a body transconductance ratio, *g_mb_*/*g_m_*, which is approximately given as ~0.2 in 0.25 µm CMOS processes. When an input signal is applied to both gate and body through capacitive coupling, *G_m_* is expected to increase by a factor of 2.4 because it consists of the sum of gate transconductance (*g_m_*) and body transconductance (*g_mb_*). This means that we can achieve the reduction in input-referred noise (IRN) by a factor of 2.4, compared to the conventional OTAs where the input signal is applied to a single NMOS or PMOS transistor. 

In this topology, the closed-loop gain is determined by the capacitive ratio between *C_IN_* and *C_F_* (or *C_IN_P,N_* and *C_F_P,N_*) as given by:

(2) $$A_{v\_QFB}=\frac{-\left(g_{m}+g_{mb}\right)r_{out}}{1+\frac{C_{F}}{C_{F}+C_{IN}}g_{m}r_{out}+\frac{C_{F\_P,N}}{C_{F\_P,N}+C_{IN\_P,N}}g_{mb}r_{out}}\approx \frac{-\left(g_{m}+g_{mb}\right)r_{out}}{1+\frac{C_{F}}{C_{F}+C_{IN}}\left(g_{m}+g_{mb}\right)r_{out}}\approx -\frac{C_{IN}}{C_{F}}$$

where *r_out_* is the resistance seen from the output nodes (*V_ON_* or *V_OP_*). To achieve a stable closed-loop gain, the cascode transistors, *M*_S1__–S4_, are added in order to boost the output impedance. The simulation result shows that an open-loop gain of >80 dB is achieved from the single stage amplifier with an aid of cascade transistors. The values of the input and feedback capacitors are carefully chosen by considering the input impedance and the strength of *g_m_* and *g_mb_* in the amplifier. Assuming the high *G_m_* of the given amplifier, the input impedance is roughly determined by the combination of *C_IN_*, *C_IN_P_,* and *C_IN_N_*. We set the total input capacitance as 15 pF to generate the input impedance of ~10 MΩ at 1 kHz (>1000 times of the electrode impedance). The rest of the capacitance is determined by considering the closed-loop gain of the preamplifier. As shown in (2), the ratio of *C_IN_* to *C_F_* is same as that of *C_IN_P_* (*C_IN_N_*) to *C_F_P_* (*C_F_N_*). The dimensions and values of the transistors and capacitors used in this preamplifier are summarized in Figure 4.

In order to save power consumption, a low-voltage power supply of 1 V is used. However, this low-voltage supply may limit the voltage swing in the output node. In particular, the cascode transistors, which are used to increase the output impedance, can limit the allowable output swing further, possibly even below 300 mV. This limited output swing sets the maximum allowable gain in the preamplifier. In our design, we chose a closed loop gain of 100 V/V to minimize any possible distortion of input signals.

### 3.2. 0.5 V-SAR ADC with Single-Clock Bootstrapping and Time-Delay Based Controller

One of the most critical blocks in the implantable neural interface is the ADC. A successive approximation register (SAR) analog-to-digital converter (ADC) has been employed to satisfy the stringent power budget. In addition, a clock/timing generator should be integrated and shared between the channels to reduce the area consumption. In this work, we use a 0.5 V supply for the ADC to minimize power consumption and isolate the supply from the analog block (1.0 V), thereby reducing the switching noise induced from the 0.5 V supply. For the multi-channel system where a dedicated SAR ADC is implemented to every channel, a synchronous SAR controller can be a good choice to reduce design complexity. However, in the synchronous SAR ADC, the clock frequency should be at least a few times higher than the sampling frequency of the ADC to generate adequate control signals. This high-frequency clock generator consumes high power and can also interfere with delicate analog signals. As an alternative, an asynchronous SAR ADC can be used. It can eliminate a high-frequency clock generator, but requires a dedicated decision circuit which may cause meta-stability and time-varying sampling rates. In addition, the asynchronous controller is hard to be shared in multi-channel systems.

**Figure 5:**
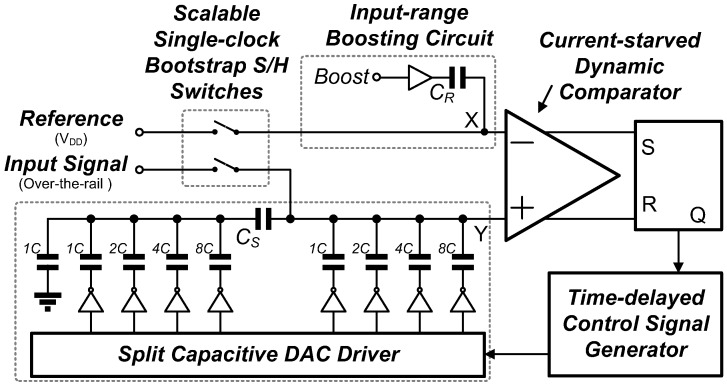
Block diagram of a 0.5 V, low-power 8-bit SAR ADC with input-range boosting, LVS bootstrapping sample/hold and time-delay control.

**Figure 6:**
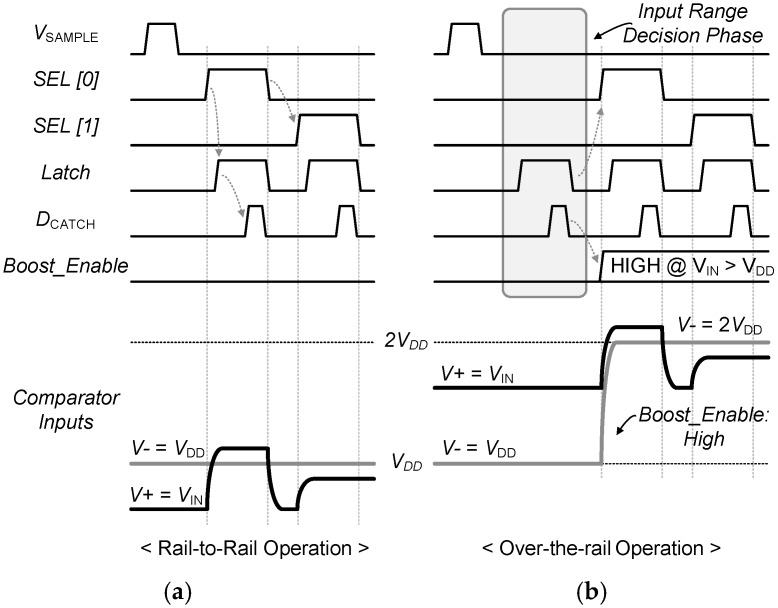
Operation of the input range boosting circuit for (**a**) rail-to-rail operation (**b**) over-the-rail operation.

With all these in considerations, we implemented a 0.5 V, 8-bit, rail-to-rail synchronous SAR ADC consisting of an input range boosting circuit block, an 8b capacitor digital to analog converter (CDAC), a low voltage single clock (LVS) bootstrapping sample and hold (S/H) circuit, a dynamic comparator, and a time-delay based controller, as shown in Figure 5 [27]. In the proposed neural interface, this ADC is interfacing between the two different power supplies: 1.0 for analog and 0.5 V for digital circuits, respectively. The input voltage range over 0.5 V is being accommodated by using the input range boosting circuit. Figure 6 explains the operation of the input range boosting circuit. The comparator in the ADC compares the input range to check whether it is higher than 0.5 V or not before the MSB conversion (SEL [0]) is initiated. If the signal is above 0.5 V, the boost signal becomes ‘high’ and the reference signal is boosted up to 2*V_DD_* (1.0 V) through *C_R_* in Figure 5. The rest of conversion is accomplished toward 2*V_DD_*. Otherwise, the conversion is achieved with a reference to the 0.5 V supply. This input range decision can boost the resolution of the ADC by one bit.

**Figure 7:**
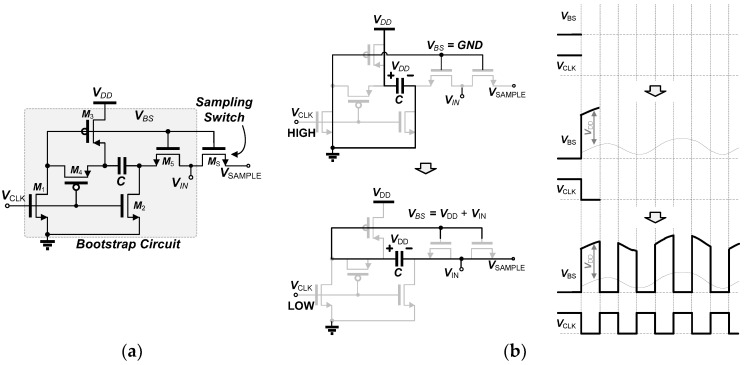
Low-power voltage-scalable single-clock (LVS) bootstrap circuit: (**a**) two-stage bootstrap circuit and (**b**) its operation and output.

The low supply voltage can save power consumption of the ADC; however, it mandates an additional function such as bootstrap sampling to compensate for performance. To realize the reliable sampling operation with the low supply voltage while minimizing nonlinearities, the LVS bootstrapping circuit, based on the voltage multiplier circuits [28], is proposed in this SAR ADC, as shown in Figure 7a. The previous bootstrapping technique requires two-phase clocks [29], resulting in high power consumption (practically, doubling the switching speed). On the contrary, the proposed LVS circuit can generate a scalable bootstrapped voltage with a true single clock, which can be expressed as:

(3) $$V_{SAMPLE}=N\times V_{DD}+V_{IN}$$

where *N* is the number of stages. The bootstrapped output signal and the operation of the LVS are illustrated in Figure 7b. During the pre-charge phase, *V_CLK_* is high, *M*_1__–3_ are ‘on’ and the capacitor, *C,* is charged to *V_DD_*, while *V_BS_* is tied to the ground. Then, during the bootstrap phase, *V_CLK_* is connected to low, *M*_4__–5_ and *M*_S_ is ‘on’ and the LVS switch can generate *V_DD_* + *V_IN_*. The output voltage of the proposed switch can be extended with additional stages for the multi-stage switching to generate a higher bootstrapped voltage. Taking advantage of low-voltage operation (0.5 V) and a high affordable voltage swing (2.5 V in 0.25 μm), the reliability can be also enhanced.

**Figure 8:**
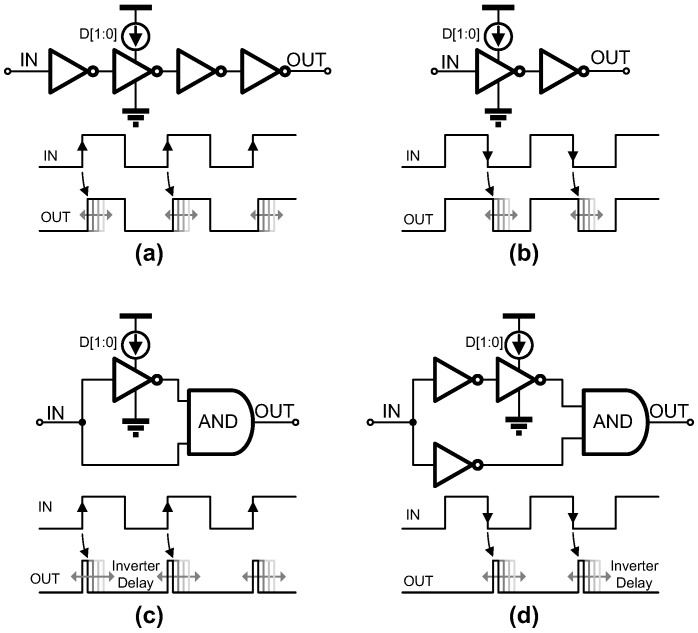
Time-delay based control units: (**a**,**b**) are the duty cycle controllers by positive and negative edge triggers, respectively, (**c**,**d**) are the one-shot generators triggered by positive and negative edges, respectively.

**Figure 9:**
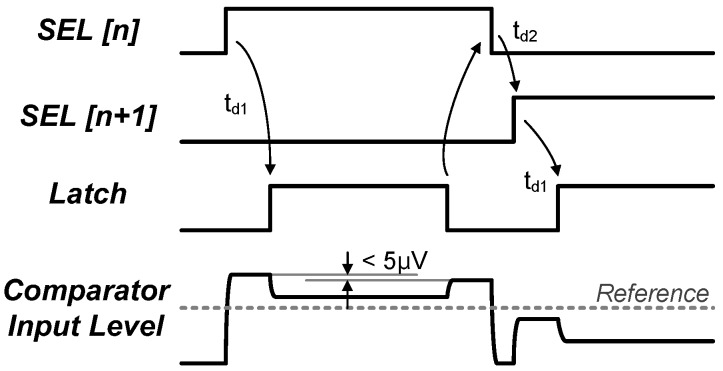
Interference-tolerant reset signal generated by the combination of the time-delay based control units.

To generate the control signals for the synchronous SAR ADC operation without a high-frequency clock, we employed a time-delayed control unit that is digitally controlled and programmed as shown in Figure 8. Even though the implemented SAR ADC operates synchronously, the high-speed clock essential for the conventional synchronous SAR ADCs is unnecessary since the proposed time-delayed units can generate any waveforms for the control of synchronous SAR ADCs. The delay units can be categorized into two types: (1) duty-cycle controllers (Figure 8a,b) and (2) one-shot signal generators (Figure 8c,d). Both types of the delay units can generate positive or negative edge-triggered signals with a programmable duty cycle in combination with logic gates. One good example for the programmability in this time-delay-based control units is an interference-tolerant reset signal which is used to prevent a kick-back effect by ensuring that the reset is completed before the capacitor-digital-to-analog-converter (CDAC) updates (the rising edge of SEL [n + 1]) as shown in Figure 9. The simulation result (Figure 9) shows that the error induced by the kick-back from the comparator by the *Latch* signal is suppressed below 5 µV. Had the conventional clocked-timing generator been used, however, complicated blocks would have been required with high-speed clock signals, thus resulting in high power consumption. In our design, the time-delayed control unit allows this control signal to be generated with much less power and reduced complexity. The measured power consumption of the control signal generator is less than 10 nW for 31.25 kS/s operation. In addition, this generator can be shared by multiple channels and significantly reduce the system complexity by eliminating all the high-frequency clocks, when compared to the conventional synchronized timing generators. For the bit decision in the ADC, a dynamic comparator having NMOS inputs and a cross-coupled degeneration has been implemented. The details of the comparator will not be given to save the length of the paper.

### 3.3. Programmable Monolithic Voltage and Current Reference

**Figure 10:**
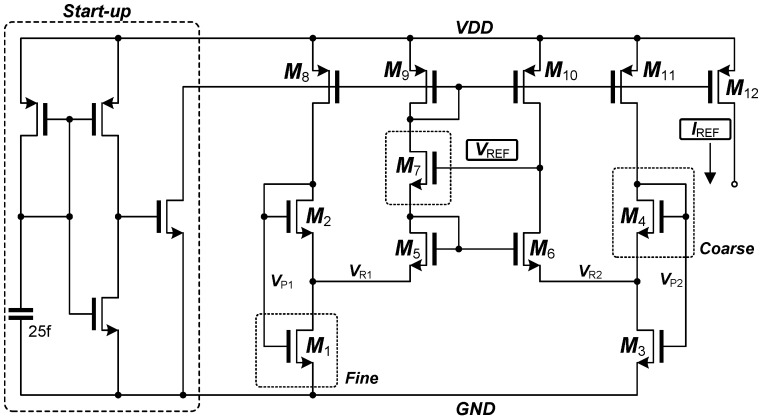
Schematic of the reference current and voltage generator.

A programmable monolithic CMOS reference voltage/current generator has been also integrated into the neural interface circuits for the completeness and reliable operations [30]. The reference generator is based on a self-cascode and beta-multiplier and operates in the input range from 1.5 V to 3.5 V which is suitable for the given battery operation. The reference generator is programmable, enabling calibration over process variations and also providing multiple reference outputs. Figure 10 depicts the schematic of the reference generator. The reference generator consists of a reference voltage/current generator and a start-up circuit. A self-cascoded pair composed of *M*_1_ and *M*_2_ operates in the strong inversion and the other self-cascoded pair composed of *M*_3_ and *M*_4_ in the weak inversion. The current mirrors are matched as 1:1. The node voltages, *V_R_*_1_ and *V_R_*_2_, are given as:

(4) $$V_{R1}\approx V_{R2}=V_{T}\ln\left(1+2S_{3}/S_{4}\right)$$

where *S*_3_ and *S*_4_ are the dimensions (W/L) of *M*_3_ and *M*_4_ and *V_T_* is the thermal voltage. The proportional-to-absolute-temperature (PTAT) reference current and the reference voltage in the implementation are given by:

(5) $$I_{REF}=I_{D2}=\frac{\beta_{2}V_{T}^{2}}{2}{\left(\frac{\ln(1+2S_{4}/S_{3})}{\sqrt{1+2S_{2}/S_{1}}-1}\right)}^{2}\;V_{REF}=V_{TH6}+V_{TH7}+V_{T}\left[ln\left(1+2S_{4}/S_{3}\right)+\ln\left(IC_{6}\right)\right]+\sqrt{\frac{2I_{REF}}{\beta_{7}}}$$

where *IC*_6_ is the inversion coefficients of *M*_6_. The generated voltage is a combination of the negative temperature coefficient of threshold voltages and the positive temperature coefficient of *V_T_*. By sizing *M*_3_ and *M*_4_ properly, a temperature insensitive voltage reference can be obtained. The voltage and current outputs of the reference generator can be precisely programmed from the digital control signals. The reference generator equips 32 and 8 taps to generate 20 to 33 nA and 0.7 to 1.0 V, respectively. The current is controlled by sizing *M*_1_–*M*_4_ (*M*_1_ for coarse and *M*_4_ for fine adjustment, respectively) and the voltage is varied by adjusting the size of *M*_7_.

### 3.4. Intra-Skin Communication for Modular Expandable Systems

Up to date, several attempts have been made to send and receive electrical signals over the human body due to their low-power operation [31,32]. The body was treated as a transmission medium: a simple conductor, a dielectric material (capacitive coupling), or a waveguide (Galvanic coupling). We decided to utilize a Galvanic coupling since the high-frequency electromagnetic waves can propagate through the body from the transmitting terminals without external wires and the surrounding environment does not significantly affect the transmission quality [31]. There are mainly two safety issues when the current is injected into the human body: (1) The maximum allowable current (300 mA) and voltage (500 V in open circuit conditions) should be limited by the Section 51.104 of the IEC 601-2-10 standard [33], and (2) the charge-injection should be balanced because the injected charge may cause the charge accumulation inside the body and result in harmful effects such as pH shift and ionic charge induction near the implanted electrodes which may damage the neural tissues.

**Figure 11:**
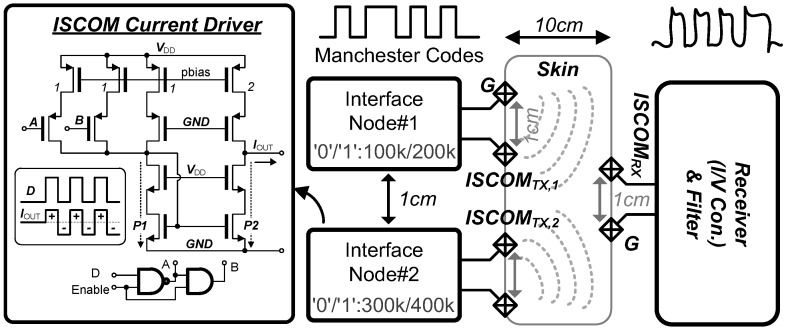
Intra-skin communication (ISCOM) configuration and current driver circuit.

We prepared a phantom brain model and conducted a few experiments with a custom PCB module which consists of V/I and I/V converters assembled using the off-the-shell components (AD620, Analog Devices Inc.) in order to set the design parameters. We found that the carrier frequency of <5 MHz can be well-balanced to provide a reasonable bit-error-rate (<10^−6^) and power consumption (~200 µW) for the ISCOM. The driver currents of <100 µA can guarantee to limit the target open circuit voltage. After the phantom brain experiments, we finalized the design of the on-chip low-power wireless ISCOM scheme as shown in Figure 11. We employed the Manchester coding technique, self-clocking, and biphasic balanced charge injection for the synchronization between the transmitter and receiver at low-power. The Manchester encoder can be easily implemented using an exclusive-OR gate with two inputs: data and clock. The encoded data is modulated by means of frequency shift keying (FSK) with a carrier frequency of 2–4 MHz. The modulated data is converted into a biphasic current by the ISCOM current driver which generates alternating currents to ensure no charge build-up inside the body. The output current polarity is determined by the current difference between the paths, *P*_1_ and *P*_2_, according to the modulated signal input. In addition, the proposed ISCOM cannot only transmit the data energy-effectively as a stand-alone system but also expand the recording coverage and sensing modalities for a distributed modular system by simply deploying multiple modules at different carrier frequencies.

## 4. Microfabricated Flexible Electrode Array

**Figure 12:**
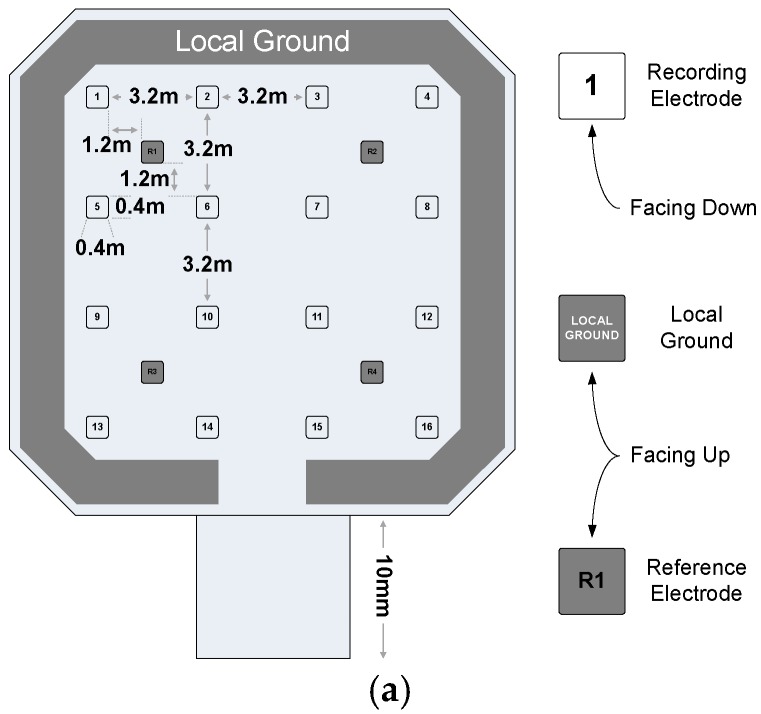

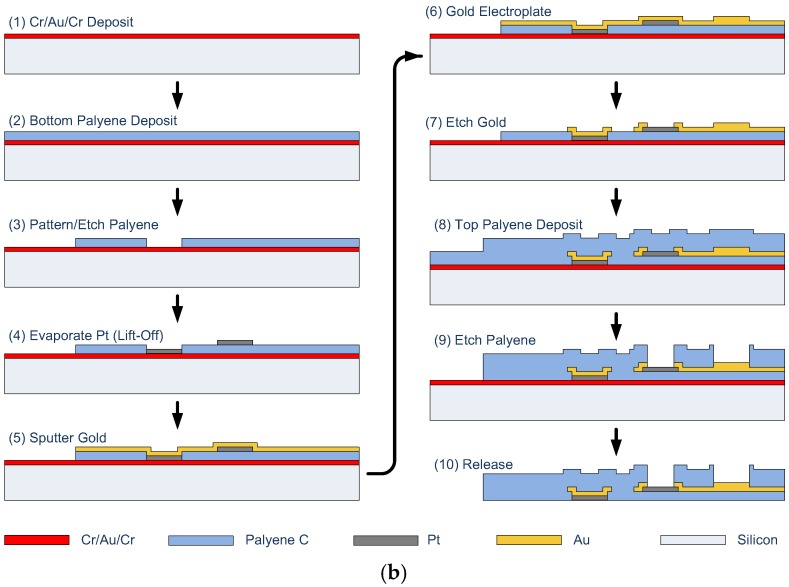
A flexible microelectrode array: (**a**) configuration (**b**) fabrication flow chart.

A thin and flexible microelectrode array designed and fabricated to be placed on top of the epidural layer in the brain. The electrodes of the device are made of Parylene C and Pt, both of which are FDA approved for bio-compatible materials. As shown in Figure 12a, there exist total 16 recording electrodes in addition to 4 reference electrodes and one large local ground electrode. The recording and reference electrodes have a diameter of ~400 μm with ~3 mm and 7 mm spacing, respectively. The thicknesses of the electrode and routing metals are defined as 0.1 µm and 0.5 µm, respectively. The recording electrodes are exposed through the bottom surface of the device to be in contact with the dura mater, while the reference and the ground electrodes are exposed on the top surface to be in contact with the surrounding average potential. 

The fabrication of the devices was performed in the Lurie Nanofabrication Facility at the University of Michigan. Simple, five-mask processes were used for fabrication. The process steps can be divided into five: (1) bottom parylene layer definition, (2) Pt electrode definition, (3) Au interconnect definition, (4) Au bonding pad definition, (5) top parylene layer definition, and (6) device release. Figure 12b illustrates the fabrication flow. First, a Cr/Au/Cr sacrificial layer for device release was deposited on a 4′′ silicon wafer using the Enerjet electron beam evaporator. A parylene layer of 12.5-μm in thickness was deposited over the sacrificial layer using the SCS PDS 2035 to form the bottom layer. This bottom parylene layer was then etched by oxygen plasma using the Plasmatherm 790 with a photoresist mask of 21-μm thick AZ 9620. Pt electrodes for the bottom and top electrodes were then deposited and lifted off. A 2.5-μm thick photoresist mask (SPR 220) was used for the lift-off mask. The 100-nm thick Pt layer was deposited using the Enerject electron beam evaporator with a Titanium adhesion layer of 10 nm and then lifted off defined in acetone. After the Pt electrode patterning, a 500 nm-thick Au layer was sputter-deposited using the Kurt J Kesker Lab 18 with a Cr adhesion layer of 5 nm. Gold was electroplated about 40 μm, selectively on the contact pads for the external connection. Then, in order to use define the interconnection, the Cr/Au seed layer was wet etched in Au etchant followed by Cr etchant with a mask defined by AZ 5214 image reversal photoresist mask of 1.8-μm in thickness. After defining the metal interconnection lines, the top parylene layer of 12.5-μm in thickness was deposited using the SCS PDS 2035. The top parylene layer was then etched using the same process for the bottom layer definition, completing the device outline and the electrode/contact pad openings. After annealing the parylene layer in a hot (>150 °C) nitrogen chamber for 24 h, the devices were finally released from the wafers by submerging in a Cr etchant to remove the sacrificial layer. Finally, the Ti adhesion layer on top of Pt electrode was removed by a quick Ti etchant dip.

## 5. Measurement Results

**Figure 13:**
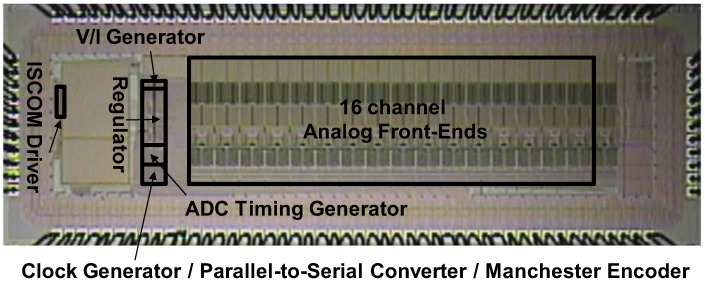
Microphotograph of the fabricated neural interface circuit chip.

The 16-channel IC for wireless monitoring of ECoG signals has been fabricated in 0.25 µm 1P5M CMOS technology and the microphotograph of the fabricated chip is shown in Figure 13. The core area of the chip, excluding I/O Pad and ESD, is 3.2 × 0.9 mm^2^ and the total power consumption is 365 µW. Actually, the net power consumption of the IC is ~200 µW and the additional power loss comes from the internal LDOs to stabilize the power supply from a rechargeable battery. This loss can be significantly reduced if the LDOs are replaced with switching power converters [34,35]. All component circuit blocks and the fabricated microelectrode array have been fully characterized at the benchtop first, followed by in-vitro experiments using the pre-recorded neural signals and then in vivo experiments in a primate in the Dan Moran Laboratory at Washington University. Table 1 summarizes the measured characteristics of the IC and the fabricated microelectrode array.

### 5.1. System Characteristics Measurement

**Figure 14:**
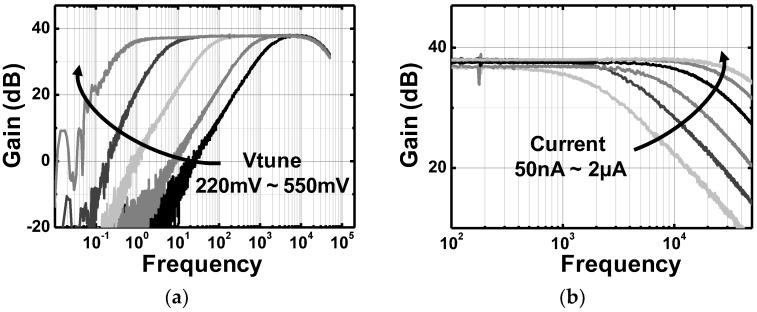
Measured frequency response of the LNA: (**a**) low frequency corner sweep and (**b**) high frequency corner sweep.

**Figure 15:**
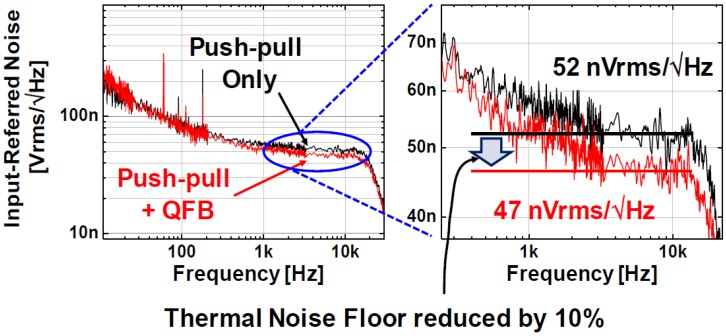
Measured power spectral densities of the input referred noise of the LNA with/without body input.

The preamplifier dissipates 0.5 µW at 1 V power supply while occupying 500 × 180 µm^2^ of the area. As shown in Figure 14, the measured mid-band gain is ~37.5 dB. The low and high corner frequencies can be digitally programmed from 0.5 Hz to 2.2 kHz and 1.5 kHz to 31 kHz, respectively, for various applications. The measured input-referred noises (IRN) are 5.62 µVrms for 10 Hz to 10 kHz and 4.26 µVrms for 1 Hz to 0.5 kHz, respectively. From the measured power consumption and IRNs, NEFs of 1.69 and 5.2 are calculated for two different frequency bands. To verify the IRN reduction by the implemented quasi-floating body gate scheme, the IRNs for both with and without the quasi-floating body gate connections are measured and compared in Figure 15. It clearly shows the thermal noise floor is reduced from 52 nV/√Hz to 47 nV/√Hz, approximately 10% reduction. Therefore, we can estimate that the proposed preamplifier architecture can effectively save ~20% of power reduction at the same noise performance.

**Figure 16:**
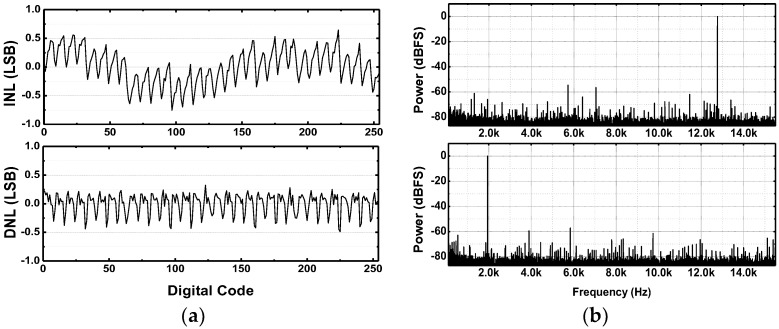
Measured SAR ADC characteristics: (**a**) INL/DNL and (**b**) FFT output spectra for 1.9474 kHz and 12.7621 kHz input signals at the sampling frequency of 31.25 kS/s.

**Figure 17:**
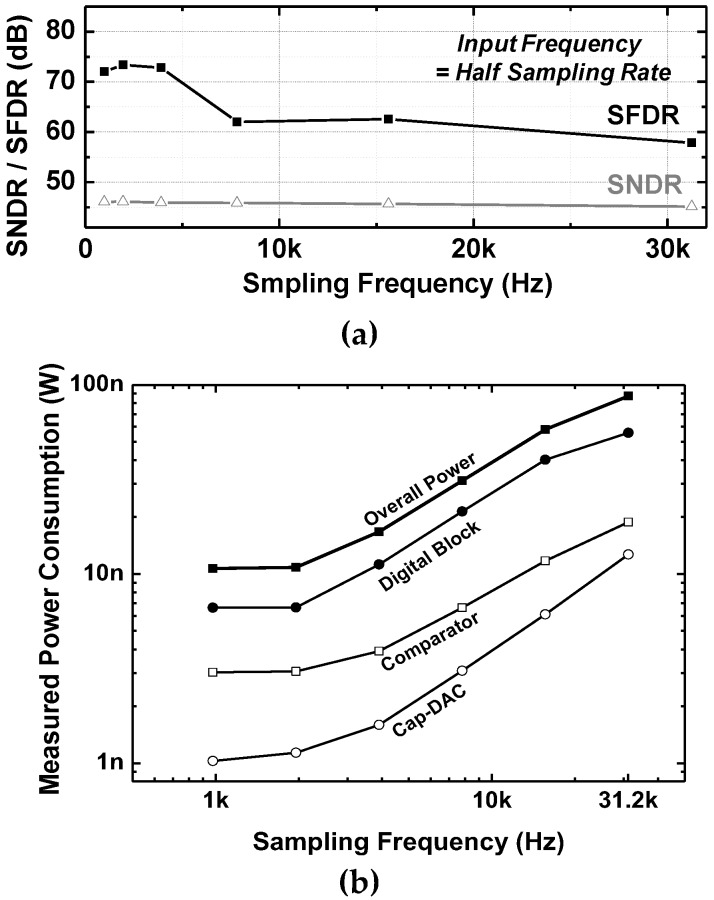
Measured SAR ADC characteristics: (**a**) SNDR/SFDR for various input and sampling frequency and (**b**) power consumption of the component blocks in the SAR ADC for 0.5 V rail-to-rail operation.

The ADC core occupies 228 × 180 µm^2^ with a unit capacitance of 49 fF. As shown in Figure 16a, the measured INL and DNL are 0.70/−0.75LSB and 0.3/−0.5LSB, respectively. The measured FFT spectrums normalized to the maximum allowable input signals of 1.9474 kHz and 12.7621 kHz at 31.25 kS/s are also shown in Figure 16b. The signal-to-noise and distortion ratio (SNDR) is 45.14 dB for the Nyquist input signal at 31.25 kS/s. Figure 17a shows the SNDR and spurious-free dynamic range (SFDR) for various sampling frequencies while maintaining the input frequencies as the half of the sampling frequency. In the low-frequency regime, especially in the ECoG band (<1 kHz), the SFDR is over 70 dB, which is suitable for ECoG recordings [36]. The measured power consumption of each individual block in the SAR ADC is also plotted in Figure 17b. The ADC consumes 87.41 nW at the maximum sampling rate of 31.25 kS/s at 0.5 V supply. The fabricated ADC shows an effective number of bit (ENOB) of 7.21 and 20 fJ/c-s in the figure of merit (FoM) for rail-to-rail operation.

**Figure 18:**
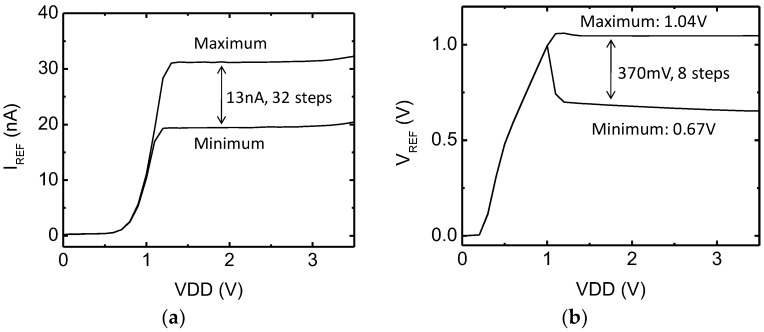
Measured outputs of the reference generator as a function of power supply voltage variations: (**a**) current reference and (**b**) voltage reference.

**Figure 19:**
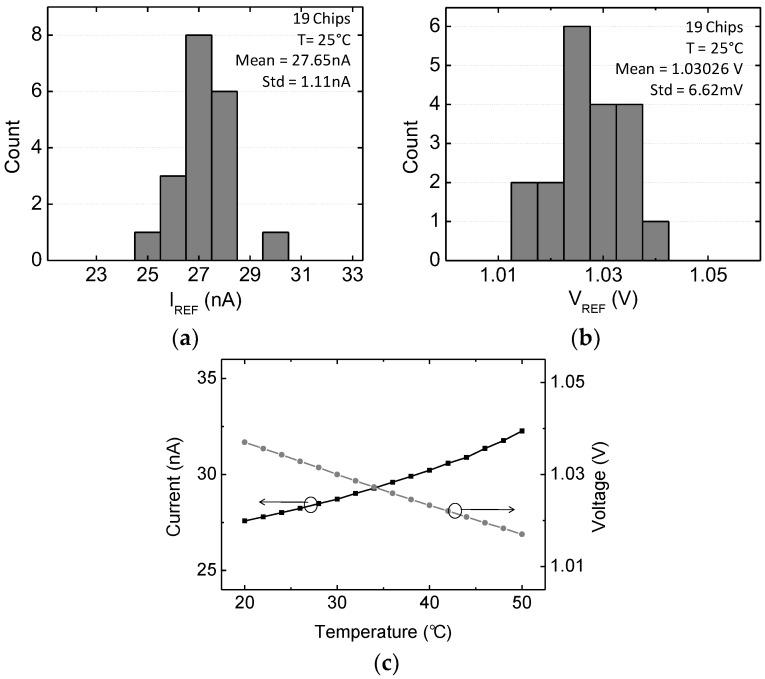
Measured variations of (**a**) reference current, and (**b**) reference voltage. (**c**) Variation of reference current and voltage as a function of temperature.

The fabricated reference generator operates from 1.2 to 3.3 V power supplies while consuming 120 nW at 1.5 V and occupying 0.011 mm^2^ in area. From the rechargeable battery output specification of 1.6 to 3.0 V, the reference generator can reliably produce the required bias. Figure 18 shows the measured variations of *I_REF_* and *V_REF_* as a function of input power supply voltage variations. It shows a linear sensitivity of 0.02%/V and 1.1%/V for voltage and current references, respectively, at the highest value. The output voltage and current references can be digitally controlled through 8 and 32 steps, respectively. The output current can be adjusted from 20 nA to 33 nA, and the output reference voltage from 0.71 V to 1.03 V. The measured reference current and voltage offset variations are 1.11 nA and 6.62 mV for 1σ variation, respectively. Figure 19 shows the process and temperature dependent variation of the reference current and voltage. The standard deviations of current and voltage among 19 chips are 1.11 nA and 6.62 mV, respectively. The measured temperature coefficient (TC) in the range of 20 °C~50 °C, which is reasonable temperature variation inside the human body, is 0.06%/C and 0.4%/C for voltage and current references, respectively. The measured power supply rejection ratio of the proposed reference generator shows <−50 dB at 100 Hz. 

**Figure 20:**
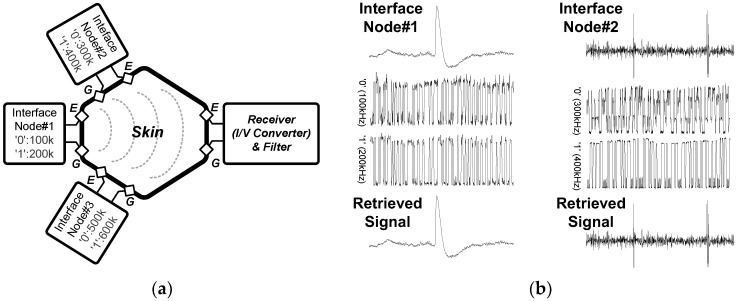
Intra-skin communication (ISCOM) characterization: (**a**) measurement diagram and (**b**) two channel data transmission.

Figure 20 shows the ISCOM measurement setup using the pre-recorded neural signals. It should be noted that the transmitted signals do not affect or interfere with neural activities because neurons (or neuronal responses) are transparent for high-frequency signals (>100 kHz). We could obtain a data bandwidth of 10 kb/s data at 160 µW and measured a channel attenuation of −17 dB at 10 cm distance from the ISCOM. For feasibility test of the distributed modular system’s expandability, two neural interface modules were placed by 1 cm apart from each other and the ISCOM_TX1_ and ISCOM_TX2_ signals from two modules were simultaneously transmitted through the skin. As shown in Figure 20b, the pre-recorded signals were transmitted through the skin and retrieved successfully. For this measurement, the current amplitude was set as ~10 µA at the ISCOM output from 1.0 V power supply. The measured bit-error-rate (BER) in this experiment was ~10^−6^.

**Figure 21:**
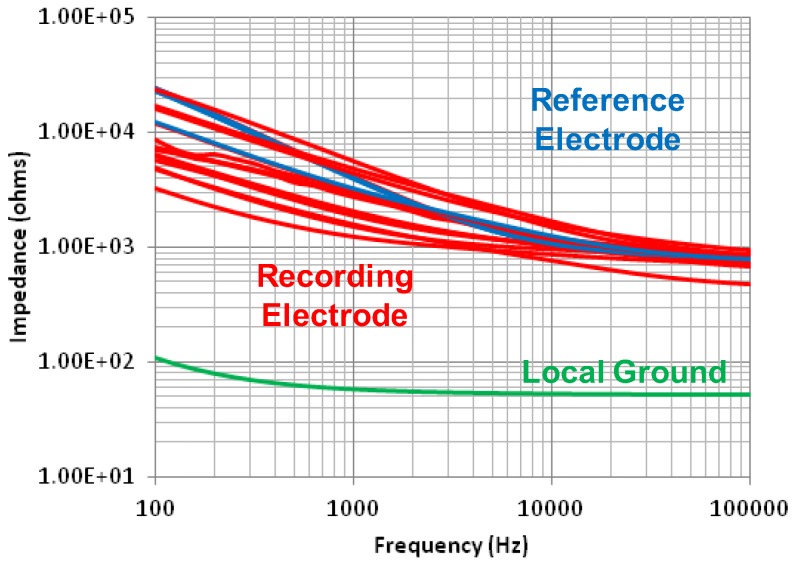
Measured impedance of the flexible microelectrode array.

Figure 21 shows the impedance of the fabricated microelectrode array. The impedance was measured in phosphate-buffered saline (PBS) solution with a Pt counter electrode by an impedance analyzer (Agilent 4192A). Due to the electrical double layer formed in series with the electrode, it shows the capacitive impedance at low frequency. The impedances of the recording and reference electrodes are distributed from ~1.2 kΩ to 5.6 kΩ at 1 kHz and that of the local ground exhibits ~60 Ω at the same frequency. Since the impedance of the recording and reference electrodes are at least 1000× smaller than that of the preamplifier, the variation of impedance does not cause serious distortion in recordings.

Table 2 summarizes the performance compared with other ECoG and EEG recording systems. This work shows the state-of-the-art power and noise performance. The NEF and PEF for 10 Hz–10 kHz are the smallest among the reported works due to the increased effective *G_m_* from the double gated push-pull amplifier. However, the gain from the increased effective *G_m_* for 1 Hz~500 Hz is rather limited. The reduced efficiency in this spectrum mainly comes from the 1/*f* noise that can be easily compensated if a noise cancellation scheme such as a chopper stabilization technique is used [22]. 

### 5.2. In Vivo Measurement

**Figure 22:**
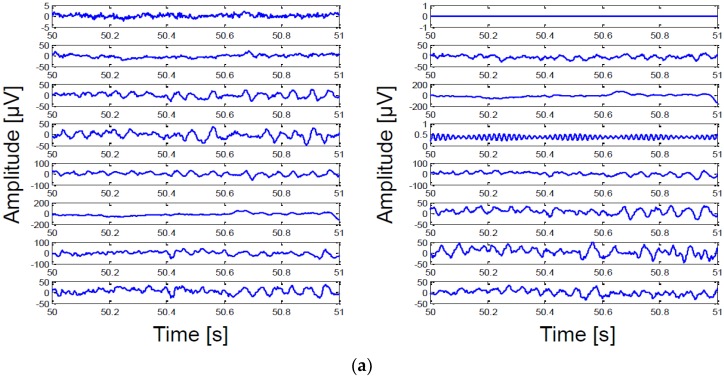

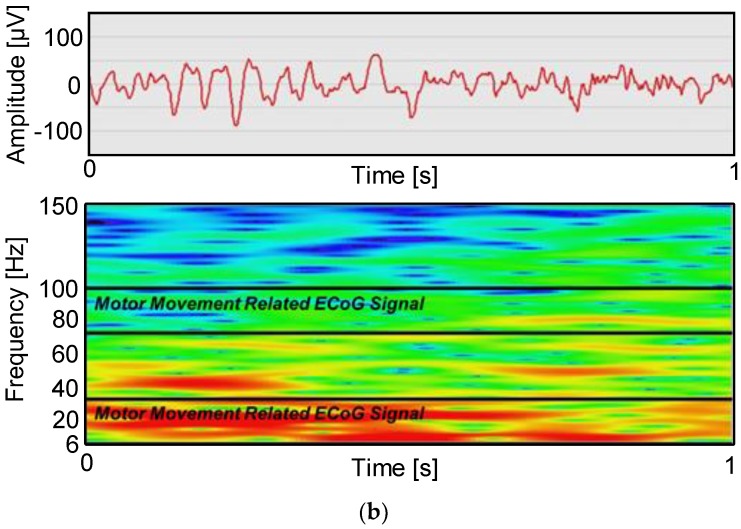
In vivo measurements: (**a**) 16-channel ECoG signals from a primate and (**b**) spectrogram of the selected single-channel from the 16-channel epidural recording.

The proposed system successfully demonstrated the in vivo measurement of epidural neural signals from a primate using our prototype interface module in the Dan Moran’s lab in Washington University. The epidural electrode array was placed on the surface of dura mater of a primate and the neural activities from 16 channels were recorded simultaneously. Figure 22a shows a 1-second waveform clip of the 16-channel ECoG signal recording. To visualize the frequency contents (α: 8–12 Hz, β: 18–26 Hz, low γ: 30–50 Hz, high γ: 70–100 Hz) of the recorded ECoG signals, a spectrogram from a selected channel with its time-domain representation is also plotted in Figure 22b. Typical motor movements are related to the ECoG neural signal power in the frequency bands of 7~30 Hz and 70~110 Hz. The spectrogram in Figure 22b shows the activity related ECoG neural signal power. 

## 6. Conclusions

We presented a minimally-invasive wireless neural interface system with the detailed description of the core IC and microelectrode array designs, and the in vivo ECoG measurement results. The interface circuits can perform 16-channel low noise, low power recording, filtering, and digitization, and data transfer through the proposed intra-skin communication (ISCOM). The interface system has been realized in an all-in-one platform by assembling the flexible microelectrode array, the signal processing core chip, the wireless data transmission module and a rechargeable battery. The platform is modular and expandable to be a distributed system over the large brain area to extend the system coverage and sensing modalities. The IC chip has been fabricated in 0.25 μm CMOS technology, and the core area of the IC is 3.2 × 0.9 mm^2^. The measured power consumption per channel is 2.5 µW and the total power consumption of the system operation from a rechargeable battery is 365 µW. The measured NEF and PEF are 1.69 and 2.86 at 10–10 kHz, respectively, and 5.2 and 27.04 at 1~500 Hz, respectively. The 16-channel flexible microelectrode array has also been fabricated by using bio-compatible Pt and Parylene-C. The impedance of the microelectrode array was measured as 1.2–5.6 kΩ, which is at least 1000× smaller than the input impedance of the interface circuits. The implemented system has successfully recorded 16-channel neural signals from a primate in vivo and transmitted the recorded data wirelessly through the skin at a rate of 10 kb/s at 160 µW power consumption with <10^−6^ of BER.

## Figures and Tables

**Table 1:** Summary of characteristics of the fabricated IC and microelectrode array.

Parameters		Measured Results
**Recording Integrated Circuit**		
Supply voltage (internally generated)		0.5/1.0 V
Supply voltage (externally supplied)		1.5–3.3 V (Li-Ion rechargeable battery)
Power/Ch.		2.5 µW
Gain		100–1000 V/V (variable)
NEF/PEF	(1~500 Hz)	5.2/27.04
	(10 Hz to 10 kHz)	1.69/2.86
Resolution		8 bit
CMRR		35 dB
PSRR		48 dB
Active area		3.2 mm × 0.9 mm (with ISCOM)
**Wireless Communication**		
Communication method		Intra-Skin Communication (ISCOM)
Supply voltage		1.0 V
Modulation		FSK
Data bandwidth		>100 kb/s
Link gain		−17 dB
Power		160 µW
Carrier frequency		2–4 MHz
Bit error rate		10^−6^
**Flexible Microelectrode Array**		
Materials		Pt on Parylene-C
Number of recording/reference channel		16/4
Impedance of electrodes @ 1 kHz		1.2–5.6 kΩ
Electrode size		400 µm in diameter
Total Area		18 mm × 18 mm

**Table 2:** Performance summary and comparison with recent ECoG and EEG works.

		[5]	[6]	[9]	[11]	[19] ^(2)^	[20] ^(2)^	This Work
Integrated Circuits	Power/Ch. (µW)	2.3	134	10.8	3.5	5	2	**2.5**
	Total Power (µW)	225	−	−	3.5	20	2	**365**
	Supply (V)	0.5	3	1.2	1	1.4–3.3	1.8	**1.0/0.5**
	N. of channel	64	64	1	1	4	1	**16**
	Channel gain (dB)	30	60	40–75	72	54–80	41,50.5	**40–60**
	Noise PSD (µV_rms_/√Hz) (nV/√Hz)	58	30 ^(1)^	85	130	100	100	**47**
	NEF	4.76	3.5	5.38	9.37	4.6	4.6	**1.69/5.86**
	PEF	11.3	42.9 ^(1)^	34.7	87.8	38.1 ^(1)^	38.1 ^(1)^	**2.86/27.04**
	CMRR (dB)	82	51	80	60	>80	100	**35**
	PSRR (dB)	75	69	60	−	>80	−	**48**
	Area/Ch. (mm^2^)	0.025	2.69	2.8	0.6	5	0.8	**0.155**
	Resolution (bit)	15	12	24 ^(3)^	12	−	−	**8**
	SNDR/SFDR (dB)	−/52	66.17/-	−/−	65.3/−	−/−	−/−	**45.14/73**
	*f*_S_ (kS/s)	−	3	−	100	−	−	**31.25**
	Wireless (Y/N)	Yes	Yes	No	No	No	No	**Yes**
	Technology (µm)	0.065	0.35	0.13	0.18	0.8	0.8	**0.25**
Electrode Array	Fabrication	Yes	Yes	No	No	No	No	**Yes**
	Diameter (µm)	260	−	−	−	−	−	**300–500**
	Impedance (kΩ)	10 @100 Hz	− ^(4)^	−	−	−	−	**1.2–5.6 @1 kHz**
	Material for electrode	Pt/Au	Pt/Ir	−	−	−	−	**Pt**

^(1)^ Estimated; ^(2)^ Analog signal processor included, no ADC; ^(3)^ an external ADC used; ^(4)^ Graphically provided.
